# The significance of inadequate transcranial Doppler studies in children with sickle cell disease

**DOI:** 10.1371/journal.pone.0181681

**Published:** 2017-07-25

**Authors:** Simon Greenwood, Colin Deane, Oliver L. Rees, Ben Freedman, Suresh Kumar, Naser Ben Ramadan, Sarah Wilkinson, Grant Marais, Julie Lord, Subarna Chakravorty, Susan E. Height, Kate Gardner, David C. Rees

**Affiliations:** 1 Vascular Laboratory, King’s College Hospital, London, United Kingdom; 2 Imperial College Medical School, London, United Kingdom; 3 Department of Paediatrics, Darent Valley Hospital, Dartford, Kent, United Kingdom; 4 Department of Paediatrics, Medway Maritime Hospital, Gillingham, Kent, United Kingdom; 5 Department of Paediatrics, Lewisham and Greenwich NHS Trust, London, United Kingdom; 6 Department of Paediatrics, Croydon University Hospital, London, United Kingdom; 7 Department of Paediatric Haematology, King’s College Hospital, London, United Kingdom; University of the West Indies Faculty of Medical Sciences Mona, JAMAICA

## Abstract

Sickle cell disease (SCD) is a common cause of cerebrovascular disease in childhood. Primary stroke prevention is effective using transcranial Doppler (TCD) scans to measure intracranial blood velocities, and regular blood transfusions or hydroxycarbamide when these are abnormal. Inadequate TCD scans occur when it is not possible to measure velocities in all the main arteries. We have investigated the prevalence and significance of this in a retrospective audit of 3915 TCD scans in 1191 children, performed between 2008 and 2015. 79% scans were normal, 6.4% conditional, 2.8% abnormal and 12% inadequate. 21.6% of 1191 patients had an inadequate scan at least once. The median age of first inadequate scan was 3.3 years (0.7–19.4), with a U-shaped frequency distribution with age: 28% aged 2–3 years, 3.5% age 10 years, 25% age 16 years. In young children reduced compliance was the main reason for inadequate TCDs, whereas in older children it was due to a poor temporal ultrasound window. The prevalence of inadequate TCD was 8% in the main Vascular Laboratory at King’s College Hospital and significantly higher at 16% in the outreach clinics (P<0.0001), probably due to the use of a portable ultrasound machine. Inadequate TCD scans were not associated with underlying cerebrovascular disease.

## Introduction

Approximately 300 000 children with sickle cell disease (SCD) are born each year[1]. Although the majority of these births are in Africa, SCD is also the most common serious monogenic condition in the USA and many European countries, including the UK and France[2]. The pathology of SCD stems from the polymerization of deoxygenated sickle hemoglobin, which results in red cell dehydration, vaso-occlusion and a cascade of secondary pathological events, including hemolysis, vascular-endothelial dysfunction, inflammation, coagulopathy and platelet activation[3]. Cerebrovascular disease is one of the most serious consequences of vasculopathy in children with SCD, and can result in acute ischemic stroke (AIS), hemorrhagic stroke or silent cerebral infarction (SCI).

AIS occurs at increased frequency in all types of SCD, although the risk is highest in those homozygous for the HbS mutation (HbSS), referred to as sickle cell anemia (SCA)[4]. It is typically associated with vasculopathy of the middle cerebral circulation, although other arteries can be affected, including the anterior cerebral[5] and extracranial carotid arteries[6]. SCI is associated with small vessel disease. Without primary prevention, the peak incidence of AIS is from 2–5 years old, with an incidence of about 1 per 100 patient years, 300 times greater than the normal population[4]. In some countries with access to safe blood transfusions, overt stroke has become less common in SCA due to primary stroke prevention[7].

The value and role of transcranial Doppler (TCD) scanning was established by Robert Adams and colleagues in the 1990s, who showed that it was possible to identify children at high risk of stroke using TCD scanning[8]. TCDs were classified based on blood velocity in the circle of Willis, expressed as time averaged mean of the maximum velocity (TAMMV). Children with abnormal, high velocities (>200cm/s) were at increased risk of stroke, which was reduced by 90% after starting regular blood transfusions[9]. TAMMVs less than 170cm/s were classified as normal with annual TCD scanning recommended, whereas velocities between 170 and 200cm/s were called conditional, and followed up more closely without starting transfusion[9]. These recommendations have been largely adopted in routine clinical practice, and have resulted in a fall in the incidence of AIS in SCD in countries able to implement them[10, 11]. Abnormal TCD velocities have also been considered an indication for hematopoietic stem cell transplantation[11], and recent evidence suggests that hydroxyurea is also an effective alternative to transfusion for primary stroke prevention[12].

Although TCD scanning has transformed the management of children with SCD, some areas of difficulty remain[13]. In some patients it is not possible to perform a satisfactory TCD scan for a variety of reasons, including difficulty cooperating with the procedure, lack of a suitable ultrasound window in the skull, aberrant anatomy in the circle of Willis, and severe underlying cerebrovascular disease. The precise definition of what constitutes an inadequate scan varies, and we have used the definition specified in the published UK standards for TCD scanning in children with SCD, which states that a scan is inadequate when it is not possible to measure blood velocities in all 10 defined arteries forming the circle of Willis (left and right middle cerebral arteries (MCAs), bifurcations, distal internal carotid arteries (dICAs), anterior cerebral arteries (ACAs) and posterior cerebral arteries (PCAs))[14]. Although only velocities measured in the MCAs, bifurcations and dICAs are used to define an abnormal scan, all velocities in all 10 segments must be measured for the result to be considered normal. This is more stringent that the definition of inadequate used in the original STOP study[9]. The significance of an inadequate scan has never been clearly defined, and causes range from trivial technical difficulties to severe vasculopathy and impending stroke. Few guidelines give clear recommendations on how to manage patients with inadequate scans at different ages. It is particularly important, because the risk of AIS is highest in children from the age of 2–5 years[4], and these young children are least able to cooperate with scanning procedures.

In view of this uncertainty, we have analysed the reasons for and the significance of inadequate TCD scans in children with SCD performed routinely between 2008 and 2015, with a view to developing an evidence-based approach to management.

## Methods

The study was performed as an audit of routinely collected clinical data, approved by the audit committee of King’s College Hospital. Patient consent was not necessary as only routinely collected data were used, all data were anonymised, and no patients were contacted as part of the study.

### Patients and clinical setting

King’s College Hospital (KCH) is involved in the care of approximately 1000 children with SCD through a network of shared-care clinics across South London and Kent. The data were collected as part of an approved audit of routinely measured clinical parameters, to assess the effectiveness of primary stroke prevention in SCD at KCH. Our routine clinical practice involves all children with SCA between the ages of 2 and 16 years having annual TCD scans. If the scan is normal, it is repeated in one year. Children with HbSC disease are routinely scanned at the ages of 2, 5 and 10 years[15]. The follow-up of inadequate scans is empirical and dependent on the age of the child. The significance of an inadequate TCD scan is clarified by magnetic resonance imaging and angiography (MRI/MRA) of the brain, although this usually requires general anesthesia in younger children (<7 years old). At King’s College Hospital, if a child under the age of 7 years has an inadequate scan, we repeat the scan every three months, and proceed to MRI/MRA after 4 inadequate scans, unless there are other neurological concerns necessitating earlier imaging. In older children, able to tolerate MRI/MRA without anesthesia, we usually proceed to MRI after two inadequate scans. In this audit, the routine clinical reports of MRI/MRAs were used regarding the presence of vasculopathy, silent cerebral infarction (SCI) and AIS.

All children scanned by the vascular laboratory at KCH between in the years 2008 to 2015 were included in this audit, including those on hydroxyurea and blood transfusion.

### TCD scanning

TCD scanning was performed in the Vascular Laboratory at KCH, and in 5 outreach clinics (Lewisham University Hospital, Queen Elizabeth Hospital Woolwich, Croydon University Hospital, Darent Valley Hospital and Medway Maritime Hospital). Accredited clinical vascular scientists with specific training in pediatric TCD scanning performed all scans. TCD imaging (TCDi) was used in all examinations, following strict protocols based on the criteria defined in the Stroke Prevention in Sickle Cell Disease study (STOP study)[9]. Early studies suggested that TCDi gave lower readings than non-imaging TCD[16], although the consensus is now that readings are similar if both methods are performed using standardised techniques[17]. Ultrasound scanners used were Acuson Sequoia, Philips iU 22 and GE Logiq e9in the KCH Vascular Laboratory and a portable Zonare Z-one for the outreach clinics, with 2–4 MHz phased array transducers and TCD application software for all systems. The TAMMVs for the MCA, ACA, PCA, bifurcation and dICA were recorded from right and left sides; the scan was classified as inadequate if velocities could not be measured in all 10 vessels for any reason[14]. The vascular scientist recorded the reason for the inadequate scan at the time of the scan. An inadequate temporal ultrasound window was reported when it was not possible to clearly visualize midbrain structures and it was not possible to identify all three ipsilateral arteries (MCA, ACA, and PCA). Reduced patient compliance was reported when children were unable to keep still or became too distressed during the scanning procedure. The third main reported cause of inadequate TCD scans was failure to identify all the vessels, despite an adequate temporal window and a cooperative child.

### Data collection and analysis

Data were collected from routine records recorded on the electronic patient record and the database of the Vascular Laboratory using a standardised form. Every TCDi report for SCD patients between August 2008 and April 2015 was analysed. Standard statistical tests were performed using Excel software (Microsoft, Redmond, WA) and SPSS version 22 (IBM, Portsmouth, UK).

## Results

### Prevalence of inadequate TCDs

Over the time course of this audit, 1191 patients with SCD underwent 3915 TCDi scans. 25% patients were taking hydroxyurea and 10% on regular blood transfusions. 49% scans were performed in the main Vascular Laboratory at King’s College Hospital, and 51% in outreach clinics using a portable machine. Of these scans, 79% were normal, 6.4% conditional, 2.8% abnormal and 12% inadequate (total of 474 inadequate scans). Scans were also classified for individual patients, with each patient being classified according to their most abnormal scan. 257 (21.6%) patients had at least one inadequate scan at some point; these patients with inadequate scans may also have had normal, conditional or abnormal scans on different occasions. 3.4% patients had an abnormal scan at some point, 6.0% a conditional scan, and 71% only normal scans. The distribution of genotypes for patients with inadequate scans was 236 (91%) HbSS, 17 (6.6%) HbSC, 2 (0.8%) HbSD-Punjab, 1 HbS/β^0^ thalassemia, 1HbS/β^+^ thalassemia; this distribution is similar to that for the overall sickle population in this study (HbSS 94%, HbSC 5%, others 1%), with no suggestion that a particular genotype was predisposed towards inadequate scans. The scan results for each patient are available in S1 File.

### Age and the prevalence of inadequate TCD scans

**Fig 1:**
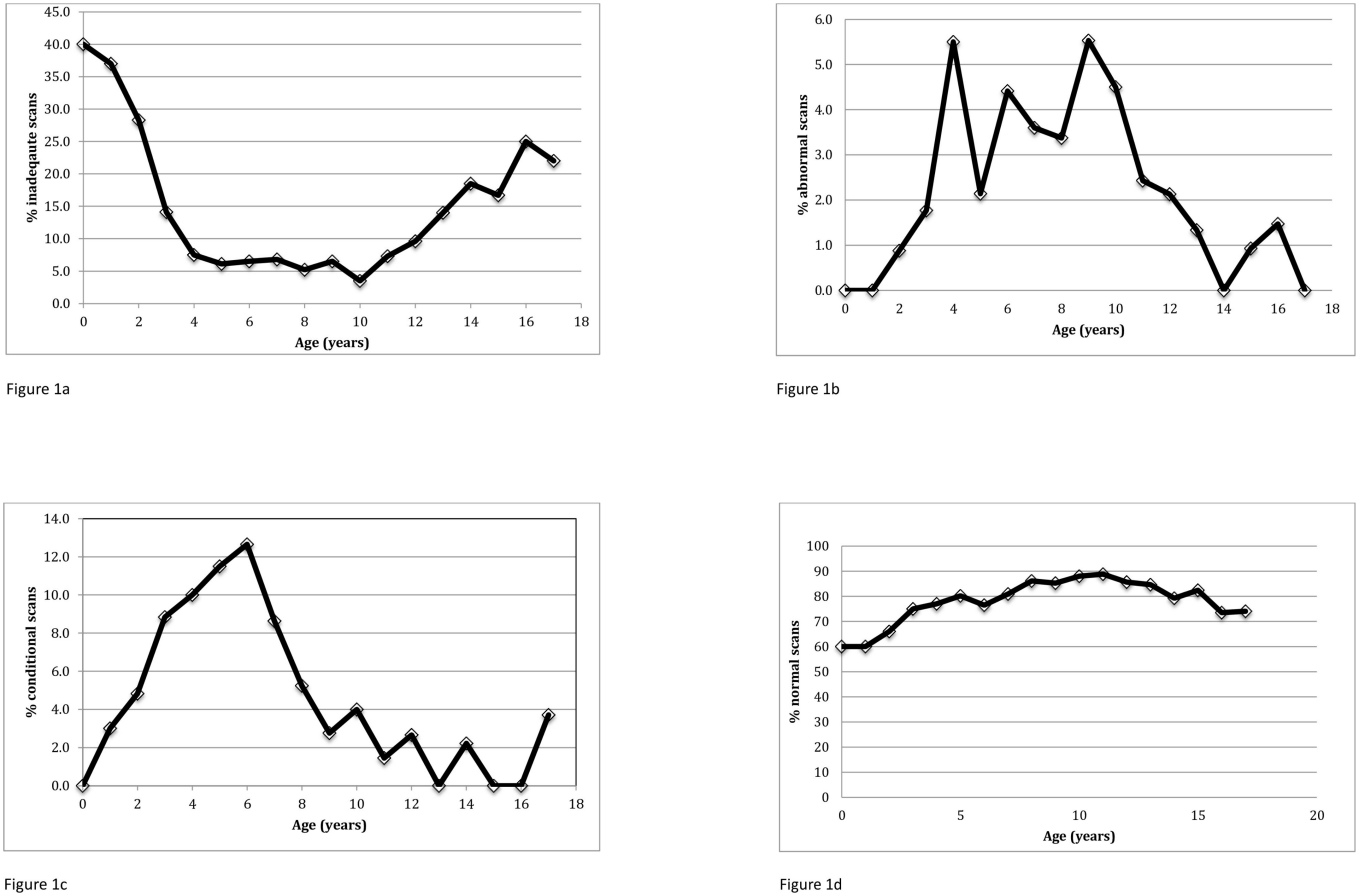
Percentage of scans in each category in different age groups, showing inadequate results (1a), abnormal velocities (1b), conditional velocities (1c) and normal results (1d).

The median age when the first inadequate scan occurred was 3.3 years (range 0.7–19.4). The total number of inadequate scans showed a bimodal distribution, with a large peak in children under the age of 5 years, and a much smaller peak in the teenage years between the ages of 13 and 14 years (Table 1). Expressed as a percentage of total scans performed at each age, inadequate TCDs showed a U-shaped distribution with age, with 28% of children between the ages of 2 and 3 having an inadequate scan, and a second peak prevalence in teenagers (Fig 1a). The lowest prevalence of inadequate scans was 3% at 10 years of age. This pattern differs from that of other scan results: the percentage of abnormal scans peaked from the ages of 4–10 years (Fig 1b), conditional scans peaked at 5 before steadily declining (Fig 1c), and normal scans were most prevalent at the ages of 10–11 (Fig 1d).

**Table 1:** Number of inadequate scans at each age, with reason for inadequacy.

Age (years)	Reason for inadequate scan					Total
	Poor temporal window	Patient compliance	All vessels not identified	Unknown	Machine limitation	
0	0	2	0	0	0	**2**
1	8	19	0	9	1	**37**
2	39	64	8	16	2	**129**
3	20	16	9	12	0	**57**
4	13	3	4	10	0	**30**
5	10	1	7	5	0	**23**
6	9	3	6	4	0	**22**
7	8	2	9	0	0	**19**
8	9	0	5	0	0	**14**
9	8	1	3	2	0	**14**
10	5	0	1	0	1	**7**
11	6	0	4	5	0	**15**
12	10	0	5	3	0	**18**
13	10	0	7	4	0	**21**
14	14	0	7	4	0	**25**
15	6	0	8	4	0	**18**
16	7	0	7	3	0	**17**
17	5	0	1	0	0	**6**
**Total**	**187**	**111**	**91**	**81**	**4**	**474**

### Causes of inadequate TCDs

**Fig 2:**
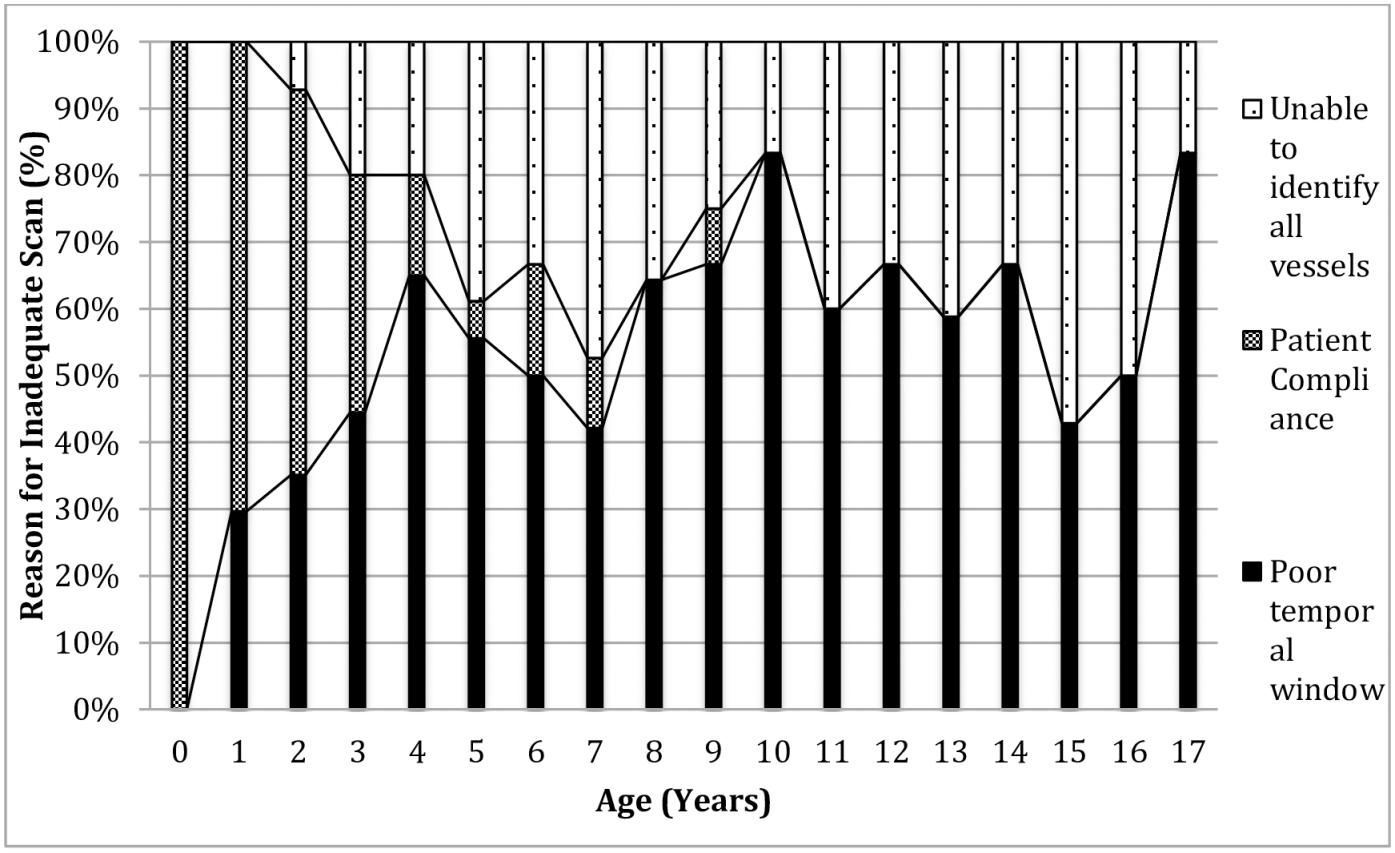
Stacked column graph showing percentage causes of inadequate TCD scans at different ages.

The reported causes of inadequate scans vary depending on the age of the child (Table 1). Overall, the most common reported cause of an inadequate scan was a poor temporal window (39%), followed by lack of patient cooperation (23%), and failure to identify all vessels (19%); in 17% cases the cause was not recorded and in 0.8% there were technical problems with the machine. Difficulty with patient cooperation was the predominant cause in young children and did not occur after the age of 9 years. Poor temporal window became relatively more important with age (Fig 2). Although specific details were not recorded, young children were either unable to keep still for long enough to allow the scan to be completed, or were frightened by the procedure. TCD scans were inadequate in 16% of the scans performed in outreach clinics, compared to 8% of those in the main KCH Vascular Laboratory (X^2^ test, P<0.001). The same staff performed the scans in both settings.

### The significance of an inadequate TCD scan

**Fig 3:**
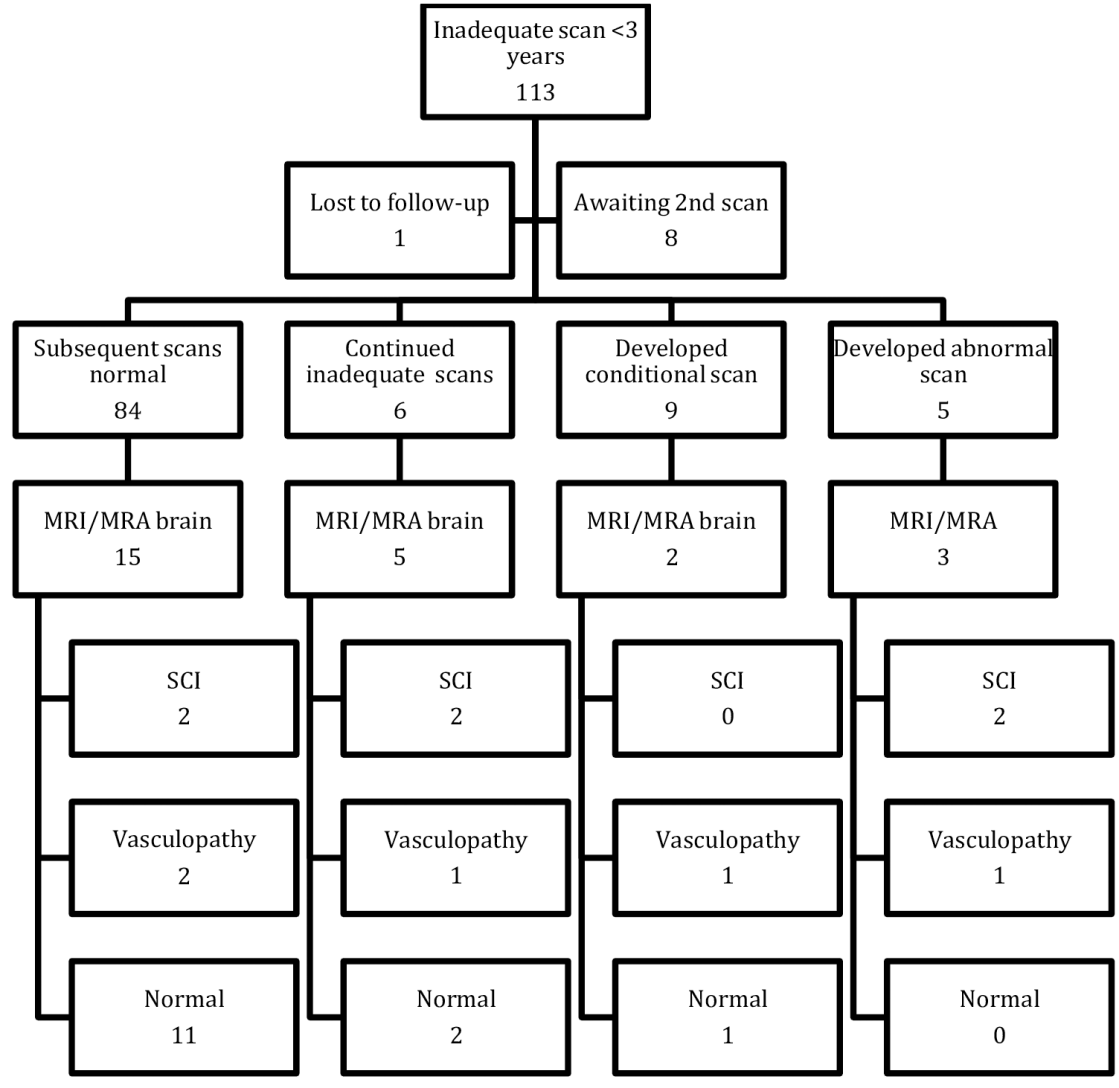
Chart showing outcomes on 113 children who had one or more inadequate TCD scan before 3 years of age. None of these children had overt strokes. SCI: silent cerebral infarction, MRI: magnetic resonance imaging, MRA: magnetic resonance angiography.

In order to clarify the predictive value of an inadequate TCD scan further, we looked in more depth at the 113 children with SCA (HbSS) who had an inadequate scan before the age of 3.0 years. We chose to focus on this group because this age coincides with the peak stroke incidence[4], and is the age when any prognostic value is most significant. The outcomes are summarized in Fig 3. To date, none of these children have gone on to have overt strokes (Median follow-up 1729 days, IQR 1009). 84 (74%) subsequently had normal TCD scans, 6 (5.3%) had persistently inadequate scans, 9 (7.9%) developed conditional velocities, and 5 (4.4%) had at least 1 abnormal scan. Of those with abnormal scans, 4 were started on regular transfusions; 1 abnormal scan was associated with acute enlargement of the spleen and exacerbation of anemia, which subsequently reverted to normal when the hemoglobin increased. Of the 113 children with inadequate scans, 25 underwent brain MRI/MRA, performed a median of 4 years after the inadequate TCD scan(range -1 to 7 years). 5 (20.0%) showed silent cerebral infarction (SCI) and 3 (12%) showed evidence of vasculopathy (stenoses, aneurysms, moyamoya) on MRA. Both these figures are in keeping with the overall levels of SCI and vasculopathy expected in an unscreened population of children with SCA.

We further divided this subgroup of under 3’s with inadequate TCD scans in to two groups: failure to assess both MCAs and ICAs (as specified in the STOP trial), and MCA/ICAs could be visualized but failure to assess the PCA/ACAs (as specified in the UK guidelines). 73 (65%) inadequate TCDs involved the MCA/ICAs and in 40 (35%) the MCA/ICAs was normal, with problems in the PCA/ACAs. In the 25 patients who had MRI scans, the type inadequate scan was not associated with vasculopathy (X^2^ = 0.000, P = 1.000), overt stroke (X^2^ = 0.694, P = 0.405) or SCIs (X^2^ = 0.146, P = 0.702) although numbers were small.

## Discussion

The prevalence and significance of inadequate TCD scans is often not considered in descriptions of cerebrovascular disease in children with SCD[18]. Studies either do not report the prevalence of inadequate TCDs[11, 19, 20], or report finding no inadequate scans[21, 22], although precise criteria for defining inadequate are usually not given. In the STOP study, 5.4% of initial TCD scans on 1934 children, aged 2–16 years, were recorded as inadequate[23]. In the BabyHug study 3.5% babies aged 7 to 17 months had inadequate TCD scans[24]. In a UK study of clinical practice, 7.9% of initial TCD scans on 542 children (median age 5 years) were inadequate[25]. We found a higher prevalence of 12% inadequate TCDs in our study, with 21.6% children having at least one inadequate scan at some point. This difference may be related to several factors: firstly, our definition of inadequate included the need to measure velocities in the ACAs and PCAs, as specified in the UK standards for TCD scanning [14], and some definitions of inadequate only include the need to assess the MCA; the number of inadequate TCDs would have been reduced by approximately 35% had we used this latter definition. Secondly, the scans in the STOP and BabyHug study were performed as part of a clinical trial, and may not relate directly to the real-world data of our study. Thirdly, the previous UK study involved a group of children with a median age of 6.25 years at first inadequate scan, compared to 3.3 years in our study, and our younger population may explain some of the difference; the prevalence of inadequate TCDs was 6.5% in our study at 6 years of age (Fig 1a). Fourthly, the previous UK study used non-imaging TCD, and it is possible that the difference in inadequacy is related to this.

The main factor associated with the increased prevalence of inadequate TCD in our study was whether the scan was performed in the main Vascular Laboratory at King’s College Hospital, or whether it was performed in one of the outreach clinics (8% versus 16%, P<0.0001). The same staff performed the scans in both settings, and it seems likely that the difference arises principally from the use of the smaller, portable scanning machine in the outreach clinics. The outreach clinic scans were also performed in a standard clinic room, as opposed to the dedicated scanning rooms available in the Vascular Laboratory. Despite the higher rate of inadequate scans, the use of the portable machine provided satisfactory results 84% of the time, and significantly reduced the number of families needing to travel to a distant hospital for TCD assessment. Following an inadequate scan in an outreach clinic, the follow up scan undertaken in either the outreach clinic or KCH vascular lab was successful in 68% of examinations.

An inadequate temporal window occurred in 187 of 3915 scans (4.8%) and accounted for 39% inadequate scans (Table 1). Previous studies suggest that inadequate temporal windows occur in 10–20% patients, although the frequency increases with age, and most of these studies involved adults[26]. In our study, it was relatively less common before the age of 4 years, but was a factor in all age groups (Fig 2). Previous studies have tried to identify risk factors for the absence of a temporal acoustic window, and in adults these have included female sex, increasing age, and black/Asian race[27]. Risk factors are less well defined in children. In some cases it was possible to use the contralateral temporal window or occipital window to overcome unilateral problems, although this depends on the experience and skill of the ultrasonographer, and on having adequate time for a more prolonged examination.

Patient compliance was the second main reason reported for an inadequate scan, occurring in 2.8% of total scans, and accounting for 23% of inadequate scans (Table 1). This was primarily related to patient age, being unusual after 3 years of age (Table 1, Fig 2). However, it is at this age that TCD scans are most important, in that the peak stroke incidence is from 2–5 years[4, 28], and the first few scans represent the best opportunity to identify cerebrovascular disease at an early stage, and to start treatment before significant vascular or brain damage has occurred. The child may be better able to tolerate the procedure in a child-friendly environment, and with appropriate distractions such as videos and mobiles. Sedation and even anesthesia may be appropriate in some circumstance, when there seems to be a high risk of significant cerebrovascular disease, although both are associated with changes in TCD velocities[29], and the results need to be interpreted cautiously.

Our study also found a relatively low prevalence of abnormal TCD velocities, with 3.4% children having a velocity >200cm/s at some point. This study was not designed to assess the prevalence of abnormal TCDs, but the lower prevalence may be due to the early use of hydroxyurea, and also the inclusion of patients with HbSC disease and other genotypes, in which TCD velocities are lower[15].

**Fig 4:**
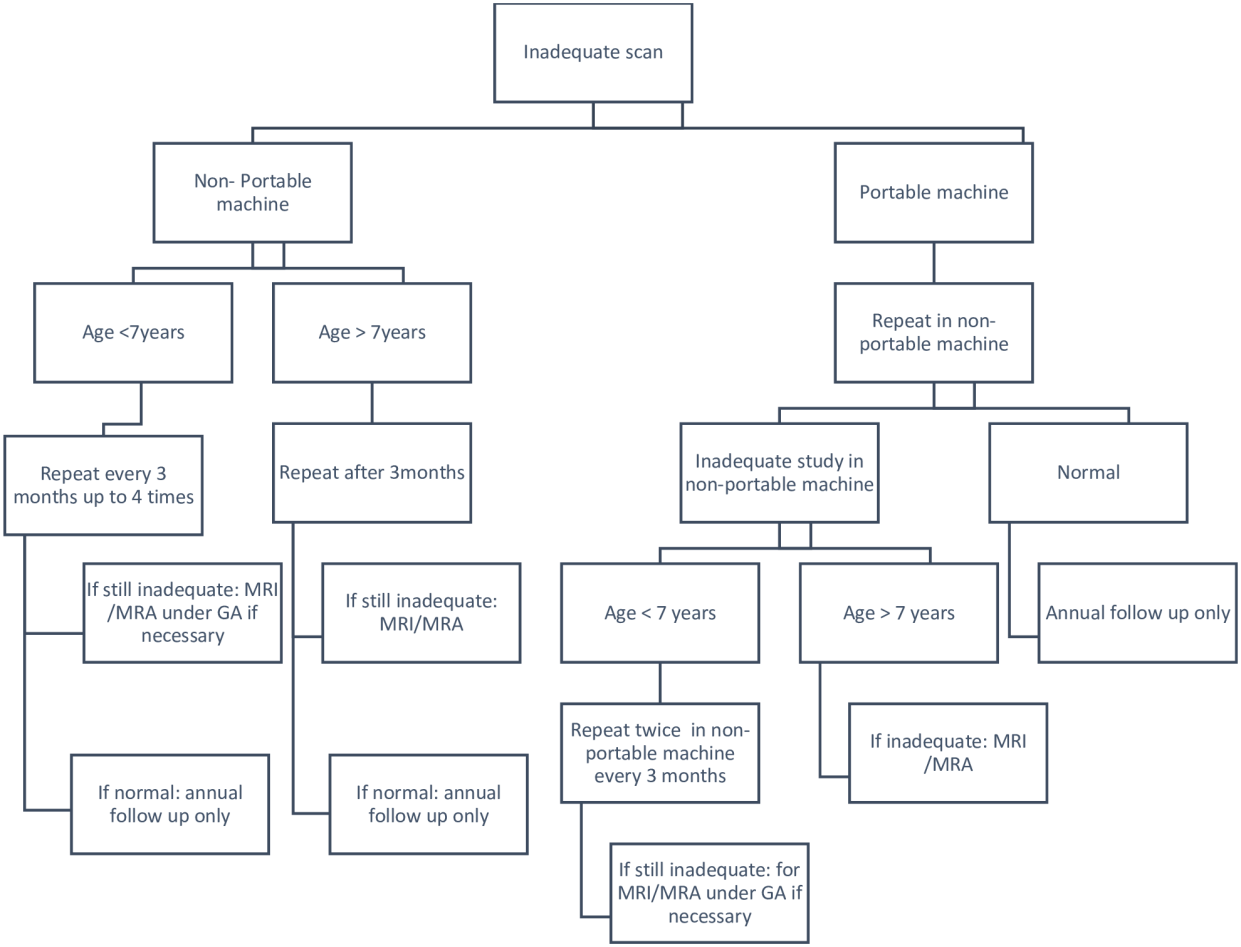
Proposed algorithm for management of children with sickle cell disease and inadequate TCD scans.

We have used the results of this audit to propose a pragmatic algorithm for the management of children with SCD and inadequate TCD scans (Fig 4). This is based on our findings that the prevalence of inadequate TCDs is significantly higher using a portable machine, and that inadequate TCDs, even in young children, are not strongly associated with cerebrovascular disease. We therefore propose that inadequate scans on a portable machine are repeated in a main vascular laboratory, and that scans should repeated over a year in young children in an attempt to get an adequate scan, before proceeding to an MRI/MRA scan. The particular issue in most children younger than 7 years is that they require sedation or general anaesthesia in order to undergo MRI, which carries significant risks. After the age of 7 years, we recommend proceeding straight to MRI/MRA after an inadequate scan because this is possible with no risk to the child.

## Conclusions

In our study, an inadequate TCD scan *per se* was not associated with an increased risk of overt stroke or cerebrovascular disease, including in those under the age of three years. In these young children, inadequate TCDs were caused by either reduced patient compliance or a poor temporal window, but did not signify severe cerebrovascular disease due to blocked or stenosed arteries. However, an inadequate scan made it more difficult to assess stroke risk and resulted in repeated scans and sometimes the need for MRI/MRA under general anesthesia. Although the highest rates of inadequate TCDs were associated with outreach clinics and a mobile TCD machine, 80% children in these clinics were adequately assessed without the need to travel to a distant hospital making this approach worthwhile. If one or more TCD scans are inadequate in an outreach clinic, it seems appropriate to repeat the scan in the central vascular laboratory before proceeding to MRI/MRA. Based on this study, we propose a pragmatic algorithm for managing children with inadequate TCD scans (Fig 4), although this may need to be modified depending on local arrangements.

## Supporting information

S1 File Inadequate data outcomes for upload. TCD results on each patient, collected as part of this audit, including details of genotype, right and left middle cerebral artery velocities, location of scan, MRI?MRA results, treatment with transfusion and hydroxyurea.
(XLSX)
